# ARQ-197, a small-molecule inhibitor of c-Met, reduces tumour burden and prevents myeloma-induced bone disease *in vivo*

**DOI:** 10.1371/journal.pone.0199517

**Published:** 2018-06-20

**Authors:** Darren L. Lath, Clive H. Buckle, Holly R. Evans, Matthew Fisher, Jenny M. Down, Michelle A. Lawson, Andrew D. Chantry

**Affiliations:** 1 Department of Oncology and Metabolism, Medical School, University of Sheffield, Sheffield, United Kingdom; 2 Mellanby Centre for Bone Research, Medical School, University of Sheffield, Sheffield, United Kingdom; 3 Department of Haematology, Sheffield Teaching Hospitals NHS Foundation Trust, Royal Hallamshire Hospital, Sheffield, United Kingdom; University of Catanzaro, ITALY

## Abstract

The receptor tyrosine kinase c-Met, its ligand HGF, and components of the downstream signalling pathway, have all been implicated in the pathogenesis of myeloma, both as modulators of plasma cell proliferation and as agents driving osteoclast differentiation and osteoblast inhibition thus, all these contribute substantially to the bone destruction typically caused by myeloma. Patients with elevated levels of HGF have a poor prognosis, therefore, targeting these entities in such patients may be of substantial benefit. We hypothesized that ARQ-197 (Tivantinib), a small molecule c-Met inhibitor, would reduce myeloma cell growth and prevent myeloma-associated bone disease in a murine model. *In vitro* we assessed the effects of ARQ-197 on myeloma cell proliferation, cytotoxicity and c-Met protein expression in human myeloma cell lines. *In vivo* we injected NOD/SCID-γ mice with PBS (non-tumour bearing) or JJN3 cells and treated them with either ARQ-197 or vehicle. *In vitro* exposure of JJN3, U266 or NCI-H929 cells to ARQ-197 resulted in a significant inhibition of cell proliferation and an induction of cell death by necrosis, probably caused by significantly reduced levels of phosphorylated c-Met. *In vivo* ARQ-197 treatment of JJN3 tumour-bearing mice resulted in a significant reduction in tumour burden, tumour cell proliferation, bone lesion number, trabecular bone loss and prevented significant decreases in the bone formation rate on the cortico-endosteal bone surface compared to the vehicle group. However, no significant differences on bone parameters were observed in non-tumour mice treated with ARQ-197 compared to vehicle, implying that in tumour-bearing mice the effects of ARQ-197 on bone cells was indirect. In summary, these res ults suggest that ARQ-197 could be a promising therapeutic in myeloma patients, leading to both a reduction in tumour burden and an inhibition of myeloma-induced bone disease.

## Introduction

Multiple myeloma (MM) is a cancer of differentiated B-cells, characterised by the accumulation of malignant plasma cells (MPCs) in the bone marrow. Common clinical manifestations include bone marrow failure leading to anaemia, impaired immunity and thrombocytopaenia, renal failure and a destructive bone disease caused by the disruption of normal bone remodelling, stimulation of osteoclastic bone resorption and inhibition of osteoblastic bone formation. Myeloma bone disease is characterised by hypercalcaemia, focal lytic lesions leading to pathological fractures, severe pain and functional deficit. Although patient survival has improved recently with the use of immunomodulatory agents, e.g. thalidomide and its analogues [1–5], proteasome inhibitors such as bortezomib [6–7] and carfilzomib [8–9], and the recent introduction of monoclonal antibodies targeting key myeloma antigens (daratumumab and elotuzumab) [10–11], the majority of patients still develop refractory disease and drug resistance [12]. Due to this, MM remains a predominantly incurable disease with patients having a median survival time of only 7 to 8 years [13]. This necessitates the need to identify new targets for drug development to reduce the tumour load and prevent further tumour-induced bone disease.

One potential group of targets for drug development are the receptor tyrosine kinases (RTKs). RTKs are transmembrane proteins that play an important role in mammalian cell processes such as cell growth, differentiation and apoptosis. Although RTKs are important in normal cell physiology and are tightly regulated, dysregulation of certain RTKs by mutation or gene rearrangement have been implicated as causative factors in the development and progression of many types of cancer [14]. The development of therapeutic agents, including low molecular weight drugs and small molecule inhibitors (SMIs) that target various RTKs, have become more prevalent, with many in various phases of clinical development [15]. In MM numerous SMIs to RTKs have been developed, many of them targeting the RTK vascular endothelial growth factor receptor. These inhibitors, when used *in vivo*, have been shown to inhibit myeloma cell proliferation, migration and survival [16–17]. However, the effect of these inhibitors on myeloma bone disease has not been thoroughly investigated.

The RTK c-Met, its natural ligand hepatocyte growth factor (HGF) and their signalling pathway (HGF/c-Met) have been implicated in the pathogenesis of MM [18–19]. HGF is expressed in human MM cell lines, promoting their growth and migration, and by freshly extracted MPCs from patient bone marrow biopsies [18–19]. HGF binds to c-Met and induces phosphorylation which leads to stimulation of cell proliferation and migration. HGF levels have been found to be elevated in the serum of myeloma patients compared to healthy matched controls, and this has correlated with a poor prognosis [20–22]. HGF has also been implicated in the pathogenesis of bone disease [23]. These authors identified HGF production by osteoclasts and expression of the HGF receptor by both osteoclasts and osteoblasts. These data suggested HGF mediated autocrine regulation of osteoclasts and paracrine regulation of osteoblasts and that HGF therefore acted as a coupling factor between osteoclast and osteoblast activity. Subsequently, a number of studies noted expression of HGF by myeloma cell lines and purified primary myeloma cells [19–20]. HGF has also been shown to inhibit BMP-induced expression of alkaline phosphatase in human mesenchymal stem cells and the murine osteoblast pre-cursor cell line C2C12 [24].

All the biological effects of HGF are mediated by c-Met and both have been found to be expressed by MPCs, again implying an autocrine loop [18,25]. Immunohistochemical staining of bone samples from myeloma patients showed concomitant expression of HGF and c-Met in MPCs. In addition, phosphorylated c-Met was observed, further demonstrating the HGF/c-Met system is active in patients with MM, and therefore this pathway could be an attractive target for novel therapeutics [26–27].

Numerous approaches have been utilised to inhibit HGF/c-Met signalling. In MM a small number of c-Met inhibitors have been investigated *in vitro* and *in vivo*. MP-470 (Amuvatanib) showed growth inhibition in the U226 human myeloma cell line and tumoricidal activity in primary MPCs without affecting normal healthy cells [28]. SU11274 inhibited myeloma cell proliferation and migration *in vitro* [25,29]. ARQ-197 (Tivantinib), a non-ATP-competitive c-Met inhibitor, induced apoptosis by >50% in 12 human myeloma cell lines, including those resistant to standard chemotherapy, and in a murine xenograft model of MM it was shown to reduce a subcutaneous tumour [30]. To date the effects of ARQ-197 on myeloma induced-bone disease have not been investigated.

In this article, we demonstrate the efficacy of ARQ-197 on the JJN3, U266 and NCI-H929 human myeloma cell lines *in vitro* and its effects *in vivo* on tumour burden and bone disease in the JJN3-NSG murine model of MM.

## Materials and methods

### Ethics statement

All procedures involving animals were approved by the Home Office (PPL 70/8799) and the University of Sheffield’s Animal Ethics Committee. Patient samples were acquired with appropriate ethical permission (REC reference: 05/Q2305/96).

### Relative HGF gene expression from human myeloma cell lines and primary sample

The myeloma cell lines JJN3, NCI-H929 and RPMI-8226 were purchased from DSMZ (Germany). U266 cells were purchased from LGC Standards (UK) and XG-1 cells were kindly provided by John Shaughnessy, Little Rock, USA. All cell lines were authenticated using polymorphic short tandem repeat (STR) loci (using the COG Cell Line and Xenograft STR Database, 8 STR loci and Amelogenin) and screened for Mycoplasma. All cell lines were seeded in 12 well plates at 2x10^5^ in 4 ml RPMI-1640 complete medium. After 48 h incubation, 2 ml used media was removed and replaced with 2 ml fresh media (containing 10% FCS, 1% penicillin/streptomycin, 100 U/100 μg/ml, 1% non-essential amino acids and 1% sodium pyruvate, 1 mM) at 37°C in 5% CO_2_. After 96 h, all cells were harvested. A bone marrow aspirate was taken from a healthy donor and primary plasma cells isolated using magnetic cell sorting with CD138^+^ Microbeads (Milenyl Biotec, Bisley, UK).Total RNA was extracted from the human myeloma and primary plasma cells using an ALLPrep isolation kit (Qiagen, Manchester, UK) followed by cDNA first strand synthesis using a Miscript RT kit (Qiagen). Real-time PCR was conducted using a human TaqMan® assay for HGF (Applied Biosystems, CA, USA), detected with ABI Prism 7900HT sequence detection system and SDS software (Life Technologies, Paisley, UK). Data were analysed using the 2 (-Delta Delta C(T)) method [31] and relative expression compared to a housekeeping gene (human GAPDH, Applied Biosystems).

### Protein expression of c-Met and phosphorylated c-Met from human myeloma cell lines

JJN3, NCI-H929, RPMI-8226, U266 and XG-1 cells were seeded in 12 well plates at 2x10^5^ in 4 ml RPMI-1640 complete medium. After 48 h incubation, 2 ml used media was removed and replaced with 2 ml fresh media. After a further 48 h, cells were harvested, pelleted (400 x g for 10 min) and resuspended in 100 μl RIPA lysis buffer (Sigma-Aldrich) containing phosphatase and protease inhibitors (1% v/v, Sigma-Aldrich). After 30 min on ice with agitation, the cell debris were pelleted at 16,000 x g for 15 min at 4°C and the supernatant containing proteins stored at -20°C until further processing. Protein samples (100 μl) were mixed with 100 μl Laemmli sample buffer (Bio-Rad, Hemel Hempstead, UK) supplemented with 5% 2-Mercaptoethanol (Sigma Aldrich) and heated for 5 min at 95°C. Samples (20 μg total protein per lane) were separated by SDS-PAGE on an 8% gel (100 v for 120 min) followed by electrophoretic transfer to a 0.45 μm PVDF transfer membrane (Millipore, Cork, IRL) using standard conditions [32]. The membrane was blocked with 5% BSA in TBS-Tween 0.05% for 1 h. The membrane was cut into strips and incubated overnight (4°C) with antibodies against c-Met (EP1454Y), phosphorylated c-Met (Tyr ^1234/1235^) and GAPDH as a housekeeper (Abcam). After a further 1 h incubation with a peroxidase-conjugated goat anti-rabbit secondary antibody (Dako, Stockport, UK), the membrane was pieced together and detection carried out with chemiluminescence (Bio-Rad, Hemel Hempstead, UK) and a ChemiDoc imaging system (Bio-Rad). Relative density was acquired with JJN3 compared against the other cell lines and all densities normalised against GAPDH.

### Cell proliferation and induced cell death of human myeloma cell lines after ARQ-197 treatment

JJN3, U266 and NCI-H929 cells were seeded in triplicate in 96 well plates at 10^4^ cells per well in 200 μl RPMI-1640 complete medium. After 48 h (start of exponential growth phase) 100 μl of used media was removed and replaced with 100 μl of fresh media containing concentrations of ARQ-197 (Stratech, Newmarket, UK) ranging from 0.1563 to 5 μM and control media containing DMSO. After a 48 h incubation, 20 μl of alamarBlue® (ThermoFisher Scientific, Altrincham, UK) was added to each well. After a further 4 h incubation, fluorescence (excitation-570 nm, emission-600 nm) was quantified using a Spectramax M5 plate reader (Molecular Devices, CA, US) and percentage reduction in cell proliferation was measured [33]. For measuring the percentage induced cell death, cells were seeded and treated (as above) and a trypan blue (Sigma-Aldrich, Gillingham, UK) exclusion count was performed [34].

### Flow cytometric analysis of induced cell death of human myeloma cell lines after ARQ-197 treatment

JJN3, U266 and NCI-H929 cells were seeded in 24 well plates at 10^5^ cells per well in 2 ml RPMI-1640 complete medium. After 48, 1 ml of used media was removed and replaced with 1 ml of fresh media containing 0.3125, 1.25 and 5 μM concentrations of ARQ-197 and control media containing DMSO. After a 48 h incubation, cells were stained with Annexin V-APC and propidium iodide (Affymetrix, CA, USA) and apoptosis/necrosis analysed by flow cytometry using a FACS calibur with Cell quest software (BD Biosciences, IL, USA) and quantification carried out using FlowJo^TM^ software (BD Biosciences).

### Protein expression of c-Met and phosphorylated c-Met from JJN3 human myeloma cells after ARQ-197 treatment

JJN3 cells were seeded in 12 well plates at 2x10^5^ in 4 ml RPMI-1640 complete medium. After 48 h incubation, 2 ml used media was removed and replaced with 2 ml fresh media. Containing either DMSO (control) or 1 μM ARQ-197. After 24 h, 48 h and 72 h cells were harvested and immunoblotted for c-Met and phospho-c-Met as described above.

### Osteoclast isolation and differentiation

Bone marrow from murine tibiae was flushed with PBS and cultured in αMEM media with 10% FCS (37°C, l 5% CO_2_) for 24 h to allow stromal cells to attach. After 24 h the cells were harvested and seeded onto sterile dentine discs (sterilised in an ultrasound bath and pre-soaked in αMEM media) in 96 well plates at 0.5 x 10^6^ cells per well in 100 μl αMEM media with 10% FCS, recombinant mouse M-CSF (150 μg/ml, R and D Systems, Oxford, UK) and recombinant mouse RANK-L (30 μg/ml, R and D Systems). After 24 h, the cells were washed (4 times with media excluding M-CSF and RANK-L) and then 200 μl fresh media containing M-CSF/RANK-L added to each well. After a further 48 h, the used media was removed and fresh media added (containing M-CSF/RANK-L) with either DMSO (N = 2), 1 μM ARQ-197 (N = 2) or 1 μM ARQ-197 with 50 ng recombinant mouse HGF (R and D systems) (N = 2). The cells were treated 3 times a week for 2 weeks. After 2 weeks the discs were fixed in 10% buffered formalin overnight and stained for tartrate-resistant acid phosphatase (TRAP) [35]. Images were taken under light microscopy.

### Treatment of JJN3 tumour-bearing and non-tumour bearing NSG mice with ARQ-197

Female NOD/SCID-γ mice (7–8 weeks old) were randomised into 3 groups (N = 8/group). Numbers per group were based on power calculations from previous studies [36]. All animals were housed in cages in pathogen free conditions and were healthy at the start of the study. Group 1 was a non-tumour bearing control group (naïve). Groups 2 and 3 were injected via the tail vein with 10^6^ JJN3 cells. From 7 to 21 days post tumour cell injection mice were treated 5 times a week by oral gavage with 100 μl 1% methylcellulose (naive, group 1 and JJN3 tumour control, group 2) or 200 mg/kg/day ARQ-197 (JJN3+ARQ-197, group 3). Calcein and alizarin complexone (both at 30 mg/kg in 0.2% sodium bicarbonate, Sigma) were given by intraperitoneal injection at the onset of treatment and then every 4 days. A supplementary study was carried out with non-tumour bearing mice treated with vehicle (N = 5) or 200 mg/kg/day ARQ-197 (N = 4) as above regimen. All treatments were carried out at the same time each day. Animals were monitored daily for any adverse effects. At the first signs of morbidity (after 3 weeks) all animals were anesthetized (100% w/v isoflurane & 2% oxygen by inhalation) for cardiac bleeding and sacrificed by cervical dislocation. Bone disease and tumour burden were assessed as described below.

### Micro-CT analysis of bone disease

Right tibiae and vertebrae (L3) were fixed in 10% formalin and scanned using a SkyScan 1272 (Bruker, Kontich, Belgium) as previously described [37]. All the measurements above followed standard guidelines [38]. Osteolytic lesions (Lesions No) were quantified using ImageJ software (v.1.47t, NIH, USA) as described previously [39].

### Histological and histomorphometric analyses of tumour burden, osteoblasts and osteoclasts

After micro-CT (μCT) analysis, the right tibiae were decalcified in EDTA at room temperature for 4 weeks, embedded longitudinally in wax and sectioned at two levels 50 um apart. 3 μm sections were stained with H/E or TRAP for tumour burden and osteoclast/osteoblast quantification respectively. Tumour burden was quantified on Haematoxylin stained sections using Osteomeasure software (Osteometrics, GA, USA) and under light microscopy using a BX53 microscope (Olympus, Southend on Sea, UK). The percentage area of JJN3 cells was determined in an area of marrow measuring 1.8 mm^2^ and with an offset of 0.25 mm from the growth plate. Osteoblast and osteoclast identification on the TRAP stained sections was carried out as previously described [37]. Left tibiae were fixed in 70% IMS, dehydrated through graded alcohols (80% - 100%) and embedded longitudinally in LR White medium resin (TAAB Laboratories, Aldermaston, UK). 10 μm sections were taken and the last pair of calcein/alizarin complexone labels analysed as previously described [37]. All the histomorphometric indices used have been standardised [40].

### Immunohistochemical analysis of cell proliferation and necrosis

Wax sections (3 μm) of the right tibiae were dewaxed in xylene, rehydrated through graded alcohols, and heat mediated antigen retrieval carried out using a water bath at 80°C for 30 min with citrate buffer pH6 (Abcam, Cambridge, UK). Endogenous peroxidase was blocked with 3% hydrogen peroxide for 30 min at room temperature (RT) and blocked in 1% casein in PBS-Tween 0.1% (TBST) for 30 min at RT. Monoclonal primary antibodies anti-human Ki-67 or anti-human Annexin V (Abcam) were added to the sections at a dilution of 1:100 in 1% casein and incubated overnight at 4°C. After 3 washes in TBST, the sections were treated with biotinylated goat anti-rabbit IgG secondary antibody (Vector Laboratories, Peterborough, UK) at 1:200 in 1% casein for 1 hr at RT. Sections were then treated with an ABC kit (Vector laboratories) for 20 min at RT and the bound antibody detected with DAB chromagen (Vector Laboratories) for 10 min at RT. The sections were washed in tap water, counter stained in Gills haematoxylin for 20 sec, dehydrated through graded alcohols, treated with xylene and mounted. The number of Ki-67 and Annexin V positive cells in a 250 x 250 μm area of tumour was quantified under light microscopy using the Osteomeasure system.

### Statistical analysis

All data sets were analysed using GraphPad Prism version 6.05 (CA, USA). Normalisation was assessed using a D’Agostino-Pearson test and subsequent parametric or non-parametric statistical tests used. If the normalisation test could not be carried out, a normal distribution was assumed. Comparisons between groups were compared using either a Student’s T-test or a One-way ANOVA with a Bonferroni post- hoc test where p<0.05 was considered significant. Data are expressed as mean ± SD.

## Results

**Fig 1:**
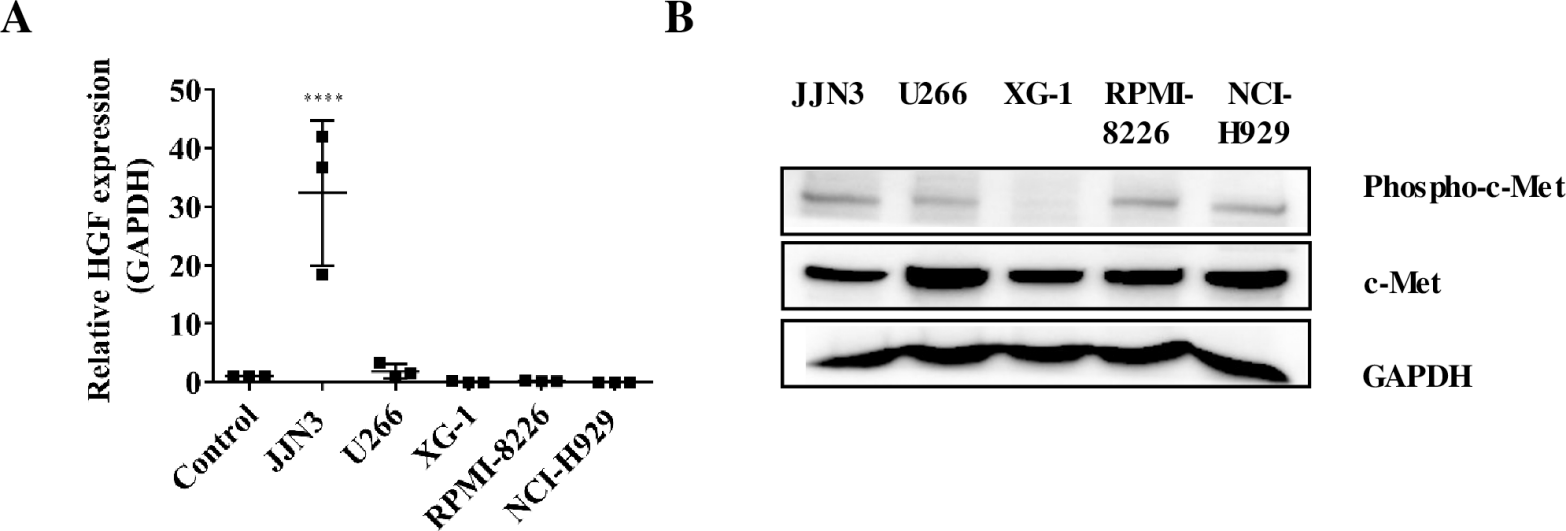
JJN3 human myeloma cells exhibited higher HGF gene expression compared to other human myeloma cell lines and healthy plasma cells and a higher phospho-c-Met protein level than other human myeloma cell lines. **(A)** Relative HGF gene expression of human myeloma cell lines JJN3, NCIH-H929, U226, XG-1 and RPMI-8226 and healthy human plasma cells (control). All data displayed as mean ± SD and analysed using a normal one-way ANOVA, where significance is indicated by ****P<0.0001. **(B)** Phospho-c-Met and total c-Met protein levels in human myeloma cells.

To find a suitable human myeloma cell line for *in vitro* assays and an *in vivo* study, we quantified the gene expression of HGF in 5 human myeloma cell lines and healthy plasma cells by real-time PCR, relative to the housekeeping gene GAPDH. JJN3 cells showed a significantly higher gene expression of HGF (p<0.0001) compared to NCI-H929, U266, XG1, RPMI-8226 cell lines and healthy human plasma cells (Fig 1A). Of the five human MM cell lines, all showed protein expression of basal c-Met with U266 cells giving the highest relative density (RD) of 1.51, with JJN3, XG-1, RPMI-8226 and NCI-H929 showing RD values of 1, 0.98, 0.99 and 1.22 respectively (Fig 1B). All of the cell lines except XG-1 cells expressed phospho-c-Met with JJN3 cells producing the highest level of expression (Relative density of 1, with U266, RPMI-8226 and NCI-H929 showing RD values of 0.82, 0.81 and 0.71 respectively (Fig 1B).

### Treatment of JJN3, U266 and NCI-H929 cells with ARQ-197 inhibits cell proliferation, induced cell death, increases cell necrosis and reduces c-Met and phosphorylated c-Met protein expression

**Fig 2:**
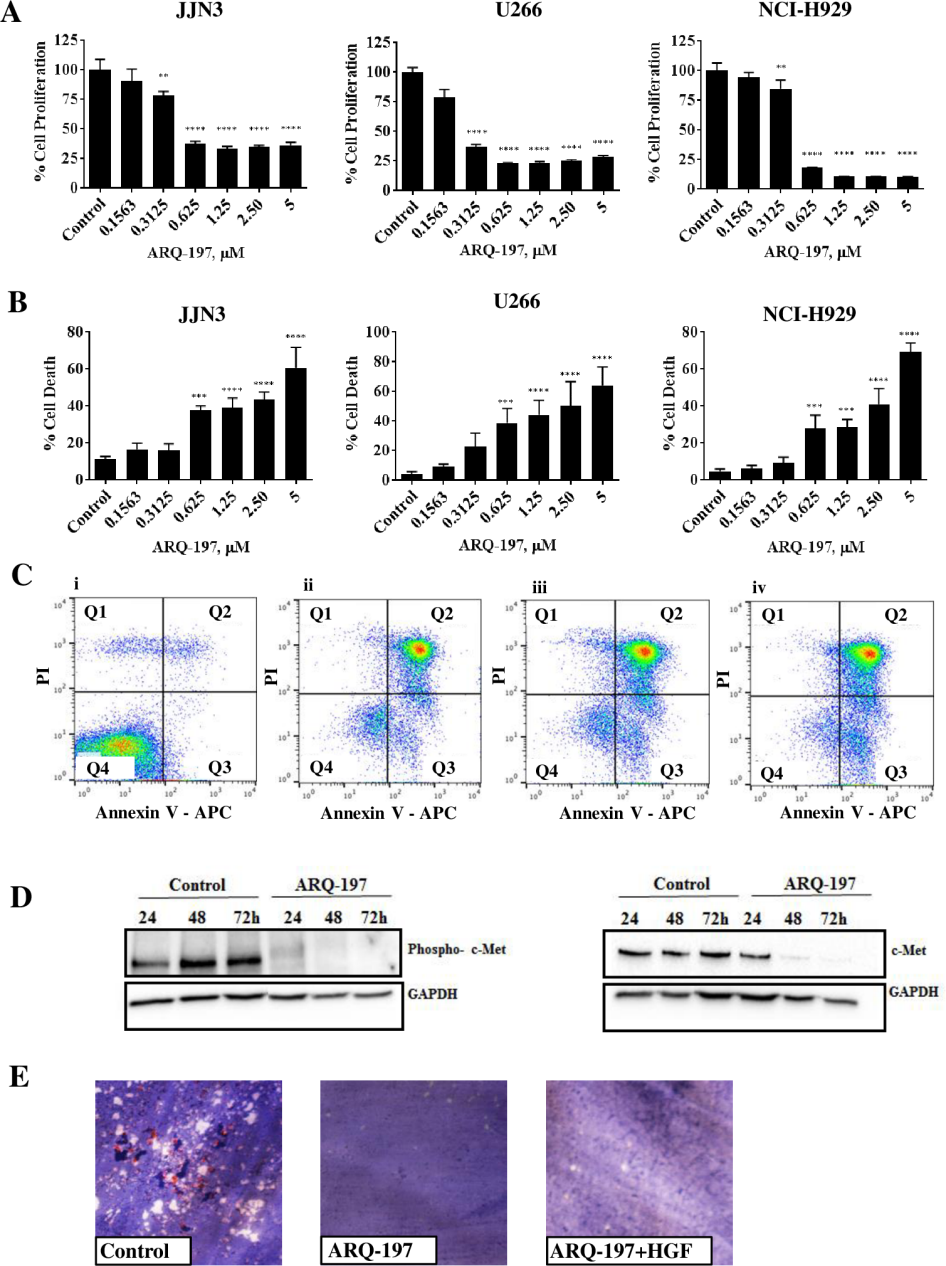
ARQ-197 inhibits cell proliferation, induces cell death by necrosis and reduces protein expression of phospho-c-Met and c-Met. (**A**) JJN3, U266 and NCI-H929 cells were incubated with control media containing DMSO or ARQ-197 at 0.1563, 0.3125, 0.625, 1.25, 2.50 or 5 μM for 48 h. Cell proliferation was measured compared to DMSO control. **(B)** JJN3, U266 and NCI-H929 cells were incubated with control media containing DMSO or ARQ-197 at 0.1563, 0.3125, 0.625, 1.25, 2.50 or 5 μM for 48 h and counted using trypan blue exclusion and the percentage cell death calculated. All data displayed as mean ± SD and analysed using a normal one-way ANOVA, where significance is indicated by **P<0.01, ***P<0.001 or ****P<0.0001. **(C)** JJN3 cells were treated with control media containing DMSO (i) or ARQ-197 at 0.3125 (ii), 1.25 (iii) or 5 μM (iv) and then stained with annexin V/PI before flow cytometric analysis. In the flow cytrometry plots, Q1 relates to dead cells, Q2 necrotic, Q3 apoptotic and Q4 viable cells. **(D)** JJN3 cells were treated with control media containing DMSO or 1 μM ARQ-197 for 24 h, 48 h and 72 h and immunoblotted with an anti-phospho-c-Met antibody and an anti-c-Met antibody. **(E)** Representative images of osteoclasts treated with either control media containing DMSO, ARQ-197 1 μM or ARQ-197 1 μM with HGF 50 ng.

To test the efficacy of ARQ-197 *in vitro*, we added varying concentrations to JJN3, U266 and NCI-H929 cells and incubated them for 48 h to assess cell proliferation and cell death (Fig 2A). Compared to the DMSO control, all concentrations of ARQ-197 except 0.1563 μM significantly reduced proliferation in all three cell lines. ARQ-197 at 0.3125 μM reduced proliferation by at least 21.5% (p<0.01) in JJN3 cells, 63% in U266 cells and 15.2% in NCI-H929 cells. Concentrations between 0.625 to 5 μM reduced proliferation by up to 67% (p<0.0001) in JJN3 cells, up to 77% (p<0.0001) in U226 cells and up to 89% (p<0.0001) in NCI-H929 cells.

Next we assessed induced cell death of JJN3, U266 and NCI-H929 cells by ARQ-197 treatment using trypan blue exclusion counts (Fig 2B). Compared to the DMSO control (11%, 4.1% and 4.7% cell death respectively), ARQ-197 at 0.1563 μM and 0.3125 μM had no significant effects in all three cell lines. Concentrations of 0.625 to 2.5 μM initiated cell death ranging from 38% - 44% (p<0.001-p<0.0001) for JJN3 cells, 39%- 50% (p<0.001-p<0.0001) for U266 cells and 28%-41% (p<0.001-p<0.0001) for NCI-H929 cells. At a concentration of 5 μM there was a more noticeable increase in cell death at 61% (p<0.0001) for JJN3 cells, 64% (p<0.0001) for U266 cells and 69% (p<0.0001) for NCI-H929 cells.

AnnexinV/PI stained JJN3 cells treated with ARQ-197 were analysed by flow cytometry to assess the quantity of viable, apoptotic and necrotic cells (Fig 2C). The majority of untreated JJN3 cells (Fig 2Ci) were viable (90.1%), with only 2.55% necrotic and 4.36% apoptotic. ARQ-197 at 0.3125 μM (Fig 2Cii) reduced the viability of JJN3 cells to 13.6% with the majority of cells necrotic (70.8%). Treatment with 1.25 μM (Fig 2Ciii) and 5 μM (Fig 2Civ) ARQ-197 showed the same levels of necrosis (71.3%). Increasing concentrations of ARQ-197 produced slightly higher numbers of apoptotic cells (12.8–18.8%). U266 and NCI-H929 cells showed similar results (not shown).

Finally, we measured c-Met and phosphorylated c-Met protein expression using immunoblotting by treating JJN3 cells with ARQ-197 at 1 μM for varying periods of time (24 h, 48 h and 72 h, Fig 2D). Compared to media controls, ARQ-197 at 1 μM visibly reduced both c-Met and phosphorylated c-Met protein expression at 24 h and almost completely inhibited expression at 48 h and 72 h.

### ARQ-197 treatment inhibits the growth of osteoclasts

The effect of ARQ-197 (1 μM) on osteoclasts seeded onto dentine discs was assessed after 14 days treatment. Compared to media controls which showed visible TRAP stained osteoclasts and erosion pits on the dentine surface (Fig 2E), treatment with ARQ-197 in the presence or absence of HGF prevented the growth of any osteoclasts and subsequent erosion pits as visualised under light microscopy (Fig 2E).

### ARQ-197 treatment reduces tumour burden and Ki-67 positive cells in JJN3-bearing NSG mice

**Fig 3:**
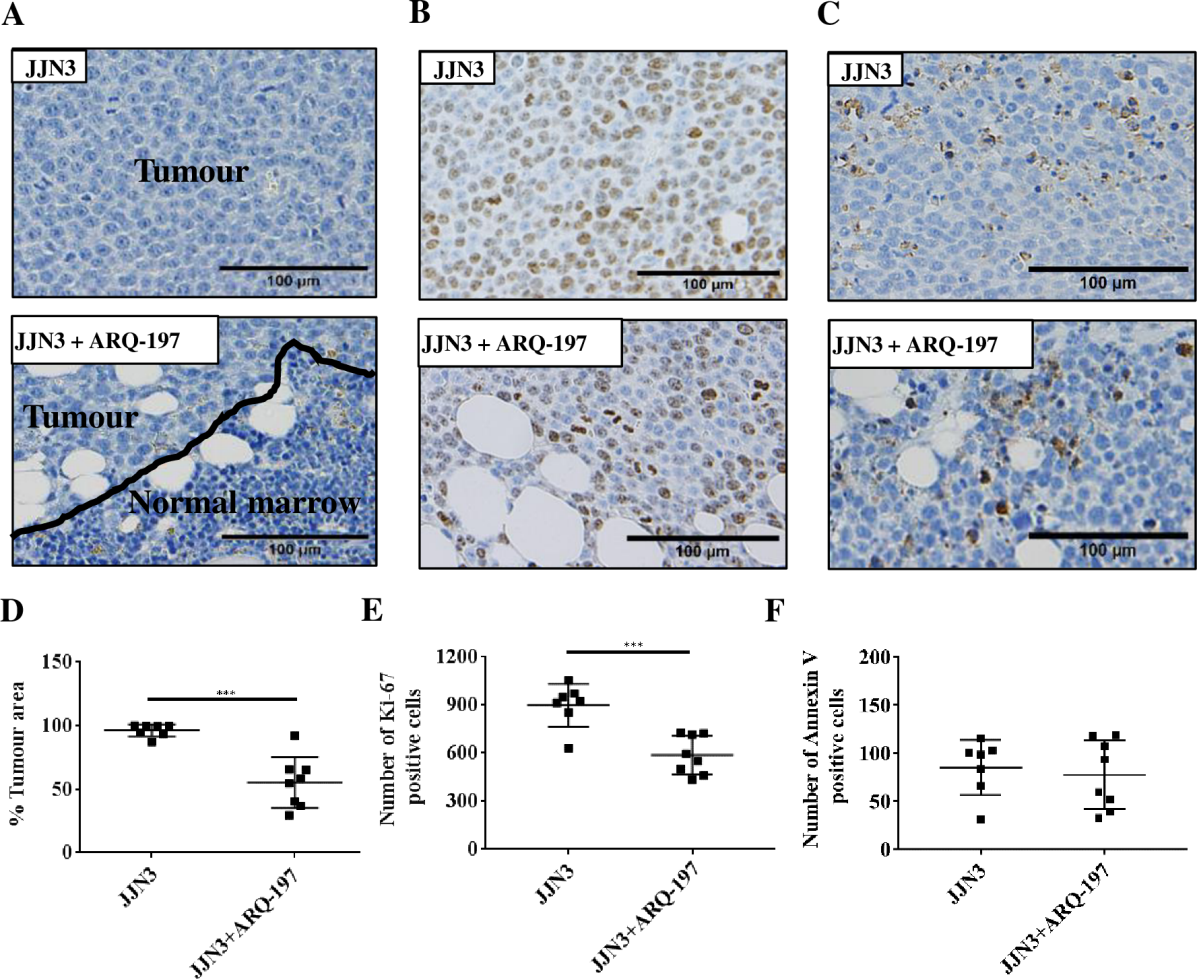
ARQ-197 reduces tumour burden and tumour cell proliferation but does not induce cell death by necrosis in JJN3 tumour-bearing mice. (**A**) Representative images of histological sections stained by Haematoxylin for tumour burden, **(B)** immunohistological staining by anti-Ki-67 or **(C)** anti-Annexin V. All scale bars = 100 μm. Histomorphometric analysis of **(D)** tumour burden, **(E)** Ki-67 and **(F)** Annexin V on tibiae from JJN3 tumour-bearing mice treated with either vehicle (JJN3) or ARQ-197 (JJN3+ARQ-197). All data displayed as mean ± SD and analysed using an unpaired t-test, where significance is indicated by ***P<0.001.

The percentage infiltration of JJN3 cells in the bone marrow of tumour-bearing mice compared to ARQ-197 treated tumour-bearing mice was analysed by histomorphometry (Fig 3A). Vehicle treated tumour-bearing control mice had 96±4.9% tumour infiltration of the bone marrow compared to 55±20% infiltration of tumour-bearing mice treated with ARQ-197, giving an overall tumour percentage reduction of approximately 43% (p<0.001, Fig 3D).

The number of Ki-67 positive tumour cells in the bone marrow was assessed by immunohistochemical staining (Fig 3B). Treatment of tumour-bearing mice with ARQ-197 significantly reduced the number of Ki-67 positive cells compared to tumour bearing mice (588±121 and 899±134 respectively, p<0.001, Fig 3E).

The number of Annexin V positive tumour cells in the bone marrow was also assessed by immunohistochemical staining (Fig 3C). There were no significant differences in Annexin V positive cells between ARQ-197 treated tumour-bearing mice and tumour-bearing mice (85±29 and 77±36 respectively, Fig 3F).

### ARQ-197 prevents tumour-induced bone disease in JJN3-NSG mice

**Fig 4:**
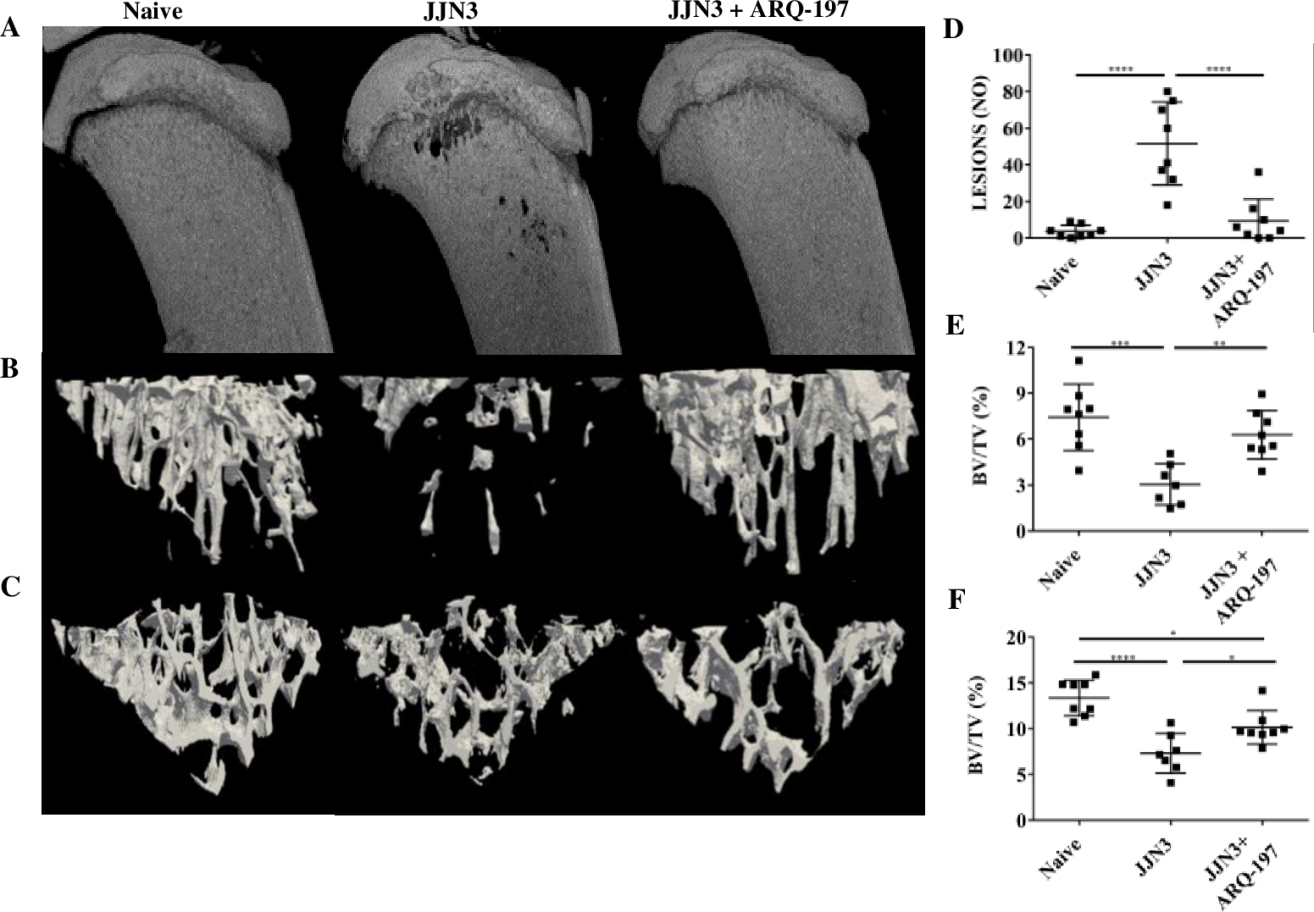
ARQ-197 reduces the number of osteolytic lesions and prevents the loss of trabecular bone in JJN3 tumour-bearing mice. **(A)** Representative μCT 3D and **(B)** cross-sectional images of tibiae or **(C)** vertebrae (L3) from naïve mice (Naïve) and tumour-bearing mice treated with vehicle (JJN3) or ARQ-197 (JJN3+ARQ-197). **(D)** Lesion counts (Lesions NO) and **(E)** trabecular bone fraction (BV/TV %) analysed by μCT of the tibiae or **(F)** vertebrae (L3) from naïve mice (Naïve) and tumour-bearing mice treated with vehicle (JJN3) or ARQ-197 (JJN3+ARQ-197). All data displayed as mean ± SD and analysed using a normal one-way ANOVA, where significance is indicated by *P<0.05, **P<0.01, ***P<0.001 or ****P<0.0001.

Micro-CT analysis of the tibiae and vertebrae from vehicle treated tumour-bearing mice revealed evidence of osteolytic lesions and a significant loss of trabecular bone compared to naïve mice (non-tumour controls) (Fig 4A–4C). In vehicle treated JJN3 tumour-bearing mice, there were visible lesions present in the tibiae (Fig 4A) compared to no visible lesions in either the naïve or tumour-bearing mice treated with ARQ-197. Automated lesion counts using 2-D software showed a significant increase in lesions in the tumour-bearing mice compared to naïve mice (51.6±8.0 and 3.6±1.2 respectively, p<0.0001, Fig 4D). Tumour-bearing mice treated with ARQ-197 showed a significantly reduced number of lesions compared with tumour-bearing mice (9.3±4.3 and 51.6±8.0 respectively, p<0.0001).

Percentage trabecular bone fraction (BV/TV, %) was significantly reduced in the tibiae of vehicle treated tumour-bearing mice compared with the naïve mice (3.0%±1.3 and 7.4%±2.2 respectively, p<0.001, Fig 4E). Tumour-bearing mice treated with ARQ-197 showed increased BV/TV compared with vehicle treated tumour-bearing mice (6.3%±1.6 and 3.0%±1.3 respectively, p<0.01, Fig 4E). BV/TV was also significantly reduced in the vertebrae of vehicle treated tumour-bearing mice compared with the naïve mice (7.3%±2.2 and 13.4%±1.9 respectively, p<0.0001, Fig 4C and 4F). Tumour-bearing mice treated with ARQ-197 showed increased vertebral BV/TV compared with vehicle treated tumour-bearing mice (10.2%±1.8 and 7.3%±2.2 respectively, p<0.05, Fig 4F). There were no significant differences in tibial or vertebral BV/TV between naïve mice and naïve mice treated with ARQ-197 (S1 Fig).

### ARQ-197 treatment reduces osteoclastic bone resorption in tumour-bearing mice

**Fig 5:**
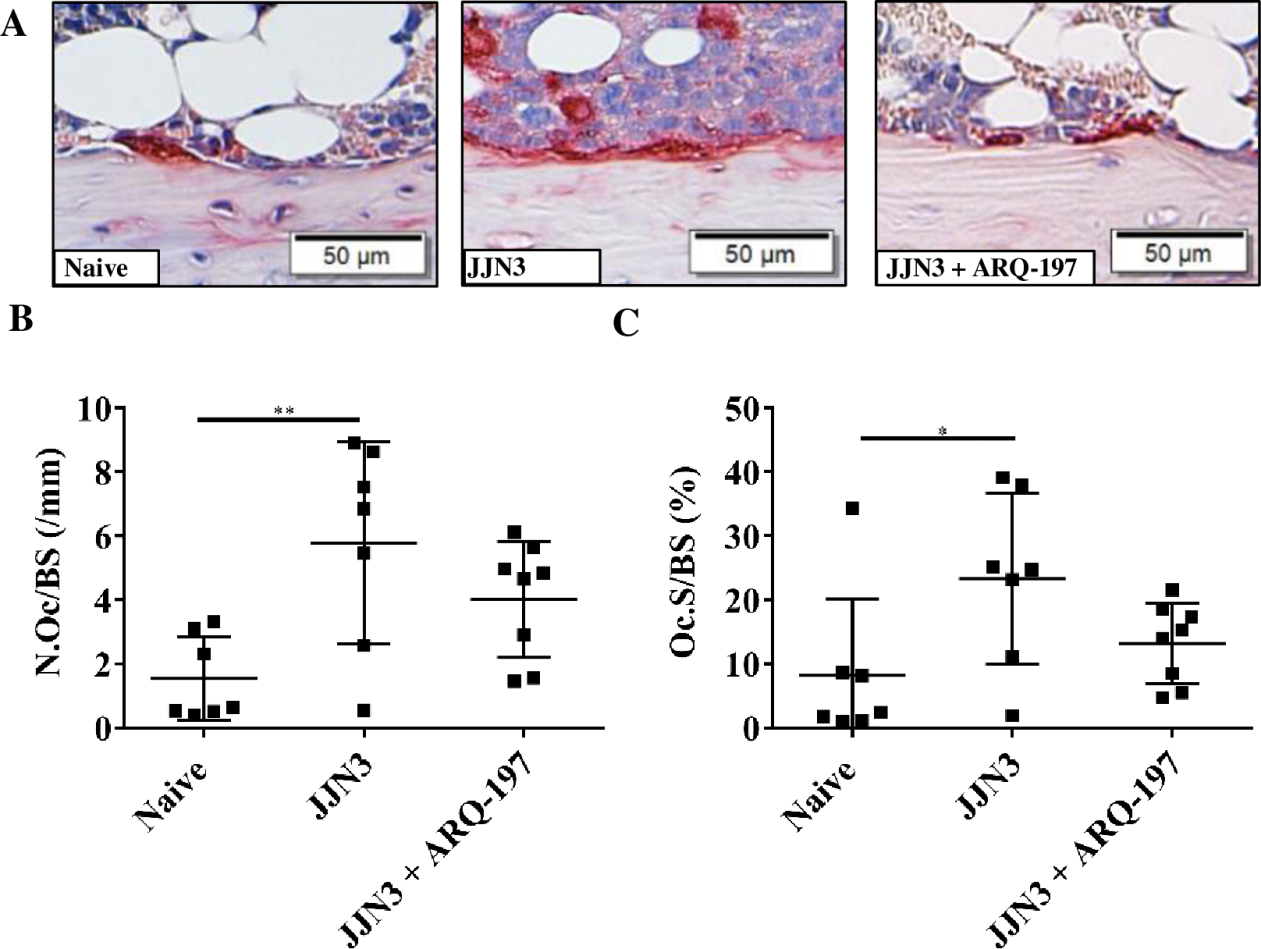
ARQ-197 reduces osteoclastic bone resorption on cortico-endosteal surfaces of the tibiae from JJN3 tumour-bearing mice. **(A)** Representative images of TRAP positive osteoclasts (stained red) on the cortico-endosteal surface of tibiae from naïve mice (Naïve) and tumour-bearing mice treated with vehicle (JJN3) or ARQ-197 (JJN3+ARQ-197) (scale bar = 50 μm). **(B)** Histomorphometric analysis of the number of TRAP positive osteoclasts per mm cortico-endosteal bone (N.Oc/BS,mm) and **(C)** the percentage coverage of TRAP positive osteoclasts on the cortico-endosteal bone (Oc.S/BS (%) from naive mice (Naïve) and tumour-bearing mice treated with vehicle (JJN3) or ARQ-197 (JJN3+ARQ-197). All data displayed as mean ± SD and analysed using a normal one-way ANOVA, where significance is indicated by *P<0.05 or **P<0.01.

Histomorphometric assessment of the cortico-endosteal surface of the tibiae showed differences in TRAP positive osteoclasts (Fig 5A). Analysis of the cortico-endosteal surfaces showed a significant increase in osteoclast number in tumour-bearing mice compared to naïve mice (5.8±3.1 and 1.5±1.3 respectively, p<0.01, Fig 5B). Treatment of tumour-bearing mice with ARQ-197 reduced osteoclast number compared to tumour-bearing mice (4.0±1.8 and 5.8±3.1 respectively, Fig 5B), although this was not significant. Percentage surface occupied by osteoclasts of the tumour-bearing mice compared to naïve mice showed a significant increase (23.3±13.4% and 8.2±12.0% respectively, p<0.05, Fig 5C). Treatment of tumour-bearing mice with ARQ-197 reduced percentage surface occupied by osteoclasts compared to tumour-bearing mice (13.20±6.3 and 23.3±13.4% respectively, Fig 5C), although this was not significant. There were no significant differences in osteoclastic bone resorption between naïve mice and naïve mice treated with ARQ-197 (S2 Fig).

### ARQ-197 treatment inhibits loss of osteoblasts in tumour-bearing mice

**Fig 6:**
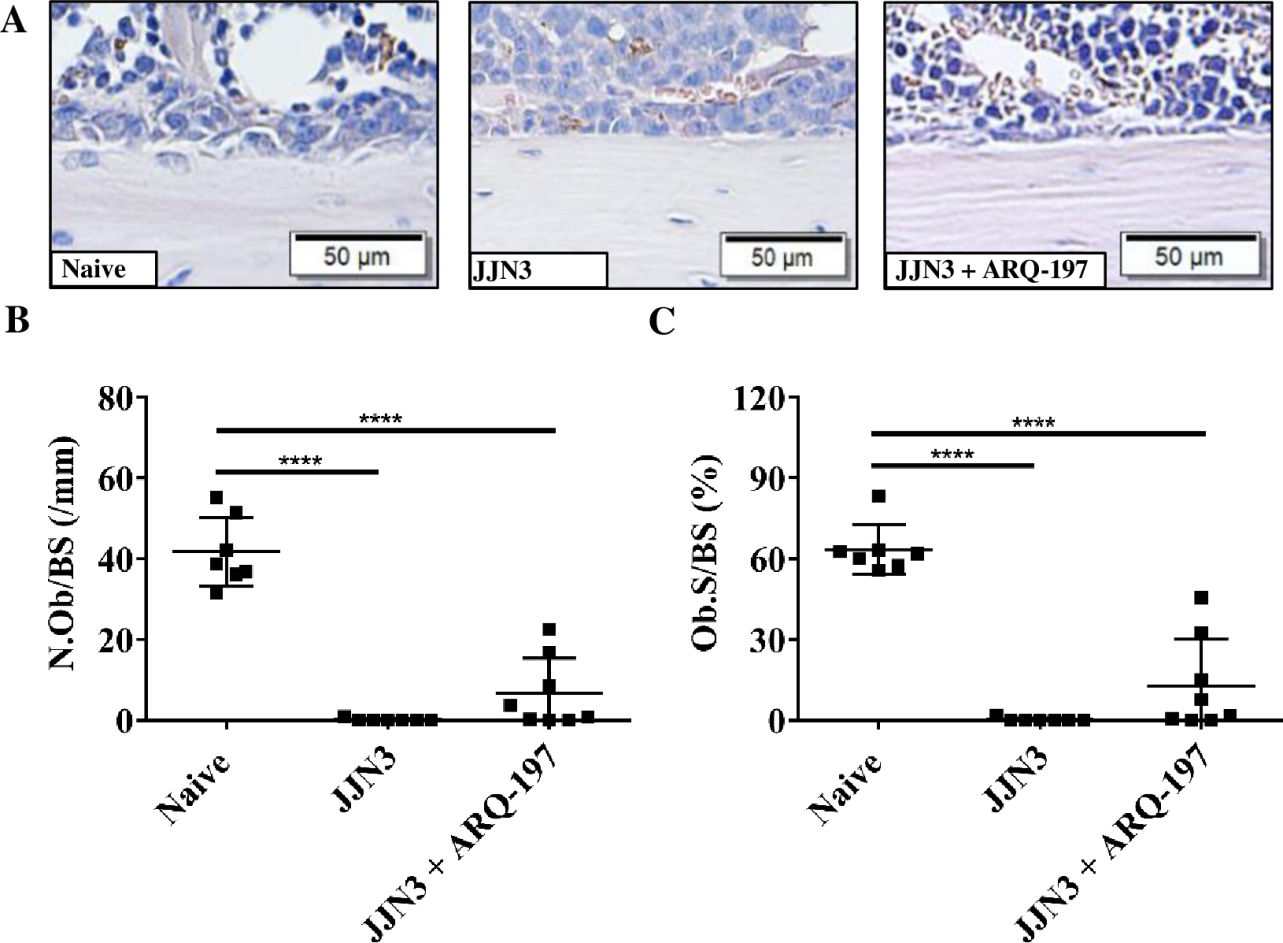
ARQ-197 inhibits osteoblast loss on the cortico-endosteal surfaces of the tibiae from JJN3 tumour-bearing mice. **(A)** Representative images of osteoblasts on the cortico-endosteal surface of tibiae from naïve mice (Naïve) and tumour-bearing mice treated with vehicle (JJN3) or ARQ-197 (JJN3+ARQ-197) (scale bar = 50 μm). **(B)** Histomorphometric analysis of the number of osteoblasts per mm cortico-endosteal bone (N.Ob/BS,mm) and **(C)** of the percentage coverage of osteoblasts on the cortico-endosteal bone (Ob.S/BS (%) from naive mice (Naïve) and tumour-bearing mice treated with vehicle (JJN3) or ARQ-197 (JJN3+ARQ-197). All data displayed as mean ± SD and analysed using a normal one-way ANOVA, where significance is indicated ****P<0.0001.

Histomorphometric assessment of the cortico-endosteal surface (Fig 6A) of tibiae showed osteoblasts identified by their unique morphology. Analysis of the cortico-endosteal surfaces showed a highly significant reduction in osteoblast number in vehicle treated tumour-bearing mice compared to naïve mice (0.1±0.3 and 41.7±8.6 respectively, p<0.0001, Fig 6B). Treatment of tumour-bearing mice with ARQ-197 increased osteoblast number slightly compared to tumour-bearing mice (6.6±8.7 and 0.1±0.3 respectively, Fig 6C), although this was not significant. Percentage surface occupied by osteoblasts of the tumour bearing mice compared to naïve mice showed a highly significant decrease (0.3±0.7% and 63.5±9.2% respectively, p<0.0001, Fig 6C). Treatment of tumour-bearing mice with ARQ-197 slightly increased percentage surface occupied by osteoblasts compared to tumour-bearing mice (12.9±17.3 and 0.3±0.7% respectively, Fig 6C), although this was not significant. There were no significant differences in osteoblast numbers between naïve mice and naïve mice treated with ARQ-197 (S3 Fig).

### ARQ-197 treatment inhibits the loss of bone formation in tumour-bearing mice

**Fig 7:**
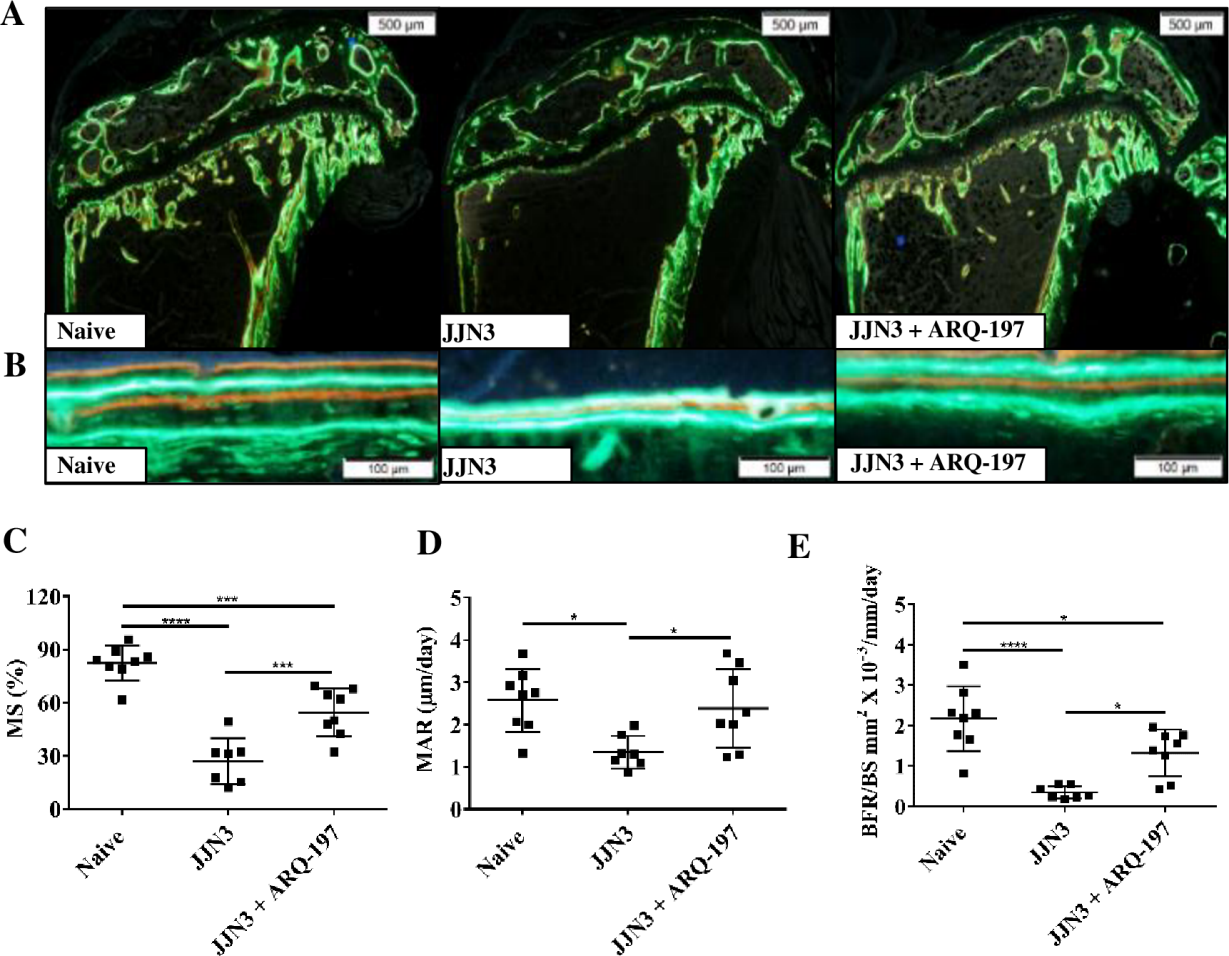
ARQ-197 inhibits bone formation loss on the cortico-endosteal surface of the tibiae from JJN3 tumour-bearing mice. Representative images of bone formation on the, cortico-endosteal surfaces, **(A)** magnification x4 (scale bar = 500 μm) and **(B)** magnification x20 (scale bar = 100 μm) of tibiae from naïve mice (Naïve) and tumour-bearing mice treated with vehicle (JJN3) or ARQ-197 (JJN3+ARQ-197). Histomorphometric analysis of **(C)** the mineralising surface (MS, %) **(D)** the mineral apposition rate (MAR, μm/day) and **(E)** the bone formation rate (BFR/BS, mm^2^ X 10^−3^/mm/day) on the cortico-endosteal bone surface of tibiae from naive mice (Naïve) and tumour-bearing mice treated with vehicle (JJN3) or ARQ-197 (JJN3+ARQ-197). All data displayed as mean ± SD and analysed using a normal one-way ANOVA, where significance is indicated by *P<0.05, ***P<0.001 or ****P<0.0001.

Histomorphometric assessment of the calcein and alizarin complexone labelling on the cortico-endosteal surface allowed assessment of bone mineralisation (Fig 7A and 7B). Mineralising surface, defined as the extent of bone surface actively mineralising, was significantly reduced in tibiae of tumour-bearing mice compared to naïve mice (27.1±13.0% and 82.4±9.9% respectively, p<0.0001, Fig 7C). Treatment of tumour-bearing mice with ARQ-197 significantly increased the extent of mineralising surface compared to tumour-bearing mice (54.5±13.5% and 27.1±13.0% respectively, p<0.001, Fig 7C). MAR, defined as distance between the labels over time, was significantly reduced in tumour-bearing mice compared to naïve mice (1.35±0.39 μm/d and 2.38±0.93 μm/d respectively, p<0.05, Fig 7D). Treatment of tumour-bearing mice with ARQ-197 significantly increased MAR (2.38±0.9 μm/d and 1.35±0.38 μm/d, p<0.05, Fig 7D). BFR was reduced in tumour-bearing mice compared to naïve mice (0.35±0.16 mm^2^ x 10^−3^/mm/d and 2.17±0.80 mm^2^ x 10^−3^/mm/d respectively, p<0.0001, Fig 7E). Treatment of tumour-bearing mice with ARQ-197 significantly increased BFR compared to tumour-bearing mice (1.32±0.57 mm^2^ x 10^−3^/mm/d and 0.35±0.16 mm^2^ x 10^−3^/mm/d, p<0.05, respectively, Fig 7E). There were no significant differences in mineralising surface, MAR or bone formation between naïve mice and naïve mice treated with ARQ-197 (S4 Fig).

## Discussion

MM is still predominately an incurable disease despite new therapies emerging [41]. The HGF/c-Met pathway has been associated with the development of numerous solid tumours [42] and has been implicated in the pathogenesis of MM with the discovery of an autocrine loop [18,29] and the pathogenesis of myeloma bone disease. Several recent studies have previously indicated a vital role for HGF in the pathogenesis of MM. In the present work we sought to assess the effect of ARQ-197, a specific c-Met inhibitor, on tumour burden and on bone disease in a xenograft model of MM. Numerous SMIs to c-Met have been tested in patients with various tumour types in clinical trials [43]. ARQ-197 is a selective inhibitor of c-Met activity, and is found to be non-ATP competitive, therefore retaining its effectiveness in cells [44]. The efficacy of this SMI has been tested on a number of human cancer cell lines, including MM [30, 45–46]. ARQ-197 has also been tested in clinical studies on numerous types of tumour including MM [47–48]. However, the effect of ARQ-197 on myeloma-induced bone disease has not yet been thoroughly assessed. Therefore, the aim of this work was to extensively assess the effects of ARQ-197 in the JJN3-NSG xenograft model of MM [36], known to develop severe bone disease, using a therapeutic approach, i.e. once tumour has been established. In addition, we also assessed the anti-tumour effects of ARQ-197 on JJN3 cells both *in vitro* and *in vivo*.

For *in vitro* and *in vivo* studies we wanted to select a suitable human MM cell line which highly expressed both HGF and phospho-c-Met. Of the five human myeloma cell lines tested, only JJN3 showed a significantly high HGF gene expression, supporting previous findings [18]. All cell lines expressed basal c-Met protein and all but one also expressed constitutively phosphorylated c-Met protein. Of these cell lines, JJN3 showed the highest protein expression of phospho-c-Met which was in agreement with previous findings [18].

Utilising the myeloma cell lines JJN3, U266 and NCI-H929 we showed that ARQ-197 reduced cell proliferation and induced cell death in a dose-dependent manner in all three cell lines, supporting previous data [30]. As the U266 cell line expressed low HGF gene expression and the NCI-H929 no HGF gene expression, this suggests that the level of HGF expression doesn’t affect the efficacy of ARQ-197 and the cytotoxic action of ARQ-197 may also be independent of c-Met status as previously suggested [47]. Subsequent Annexin V/PI staining of ARQ-197 treated JJN3 cells showed cell death as a result of necrosis rather than apoptosis, which suggests ARQ-197 has a cytotoxic effect on JJN3 cells. Immunoblotting of JJN3 cell lysates showed that treatment by ARQ-197 reduced phospho-c-Met (Y1234/1235) and c-Met (EP1454Y) protein expression, consistent with previous findings using human myeloma cell lines [30].

The effect of ARQ-197 treatment on osteoclasts *in vitro* was also assessed. After two weeks treatment, there were no visible signs of osteoclasts or their activity as indicated by dentine erosion. This indicates that osteoclasts *in vitro* are highly sensitive to the effects of ARQ-197, either having a cytotoxic effect on osteoclasts or inhibiting proliferation by blocking c-Met phosphorylation. The addition of HGF did not alter the effects of ARQ-197 on osteoclasts.

Next we assessed the effect of ARQ-197 in a xenograft model of MM, where NSG mice were injected with human JJN3 cells, resulting in tumour infiltration of the bone marrow and subsequent tumour growth and development of lytic bone disease. Treatment of tumour-bearing mice with ARQ-197 resulted in a 43% reduction in tumour burden compared to those treated with vehicle. Although this tumour reduction was significant, when JJN3 cells were treated with ARQ-197 *in vitro* the reduction in cell proliferation was more pronounced. This variance could arise from the tumour cells growing independent of c-Met signalling, suggesting ARQ-197 would be less effective as hypothesised in a previous study [30]. Alternatively, the concentration of ARQ-197 in the bone marrow is maybe lower than *in vitro*. Therefore, potentially higher or more frequent doses may be required to increase its anti-tumour effects *in vivo*. Previous studies on the anti-tumour effects of ARQ-197 in murine xenograft models have shown a reduction in tumour volume [30,45]. Similarly, the effects of ARQ-197 in human clinical trials have been encouraging and have been shown to stabilise disease in some patients [48–50]. Treatment of tumour-bearing mice with ARQ-197 resulted in a significantly reduced number of Ki-67 positive tumour cells but had no effect on the number of Annexin V positive tumour cells. This suggests that the reduction in tumour burden is as a consequence of reduced tumour cell proliferation rather than cell death by necrosis.

The effect of ARQ-197 treatment on tumour-induced bone disease was next assessed. Although the reduction in tumour burden was not as effective as expected, the effects on preventing myeloma-induced bone disease *in vivo* were quite pronounced, especially in a murine model of myeloma with aggressive disease and significant bone loss at the end stages [35]. Tumour-bearing mice treated with ARQ-197, compared to vehicle treated control mice, resulted in an inhibition of tumour-induced bone loss of trabecular bone. Since JJN3 cells secrete high levels of the osteoblast inhibitory factor HGF, a reduction in tumour volume may prevent the development of bone disease. ARQ-197 treatment also significantly reduced the number of osteolytic lesions compared to tumour-bearing mice. Histologically, tumour-bearing mice had increased osteoclast numbers and decreased osteoblast numbers. This was probably as a direct effect of increased tumour volume in these mice, as myeloma tumour cells secrete numerous osteoclast activating factors [51] and osteoblast inhibitory factors, including HGF [52]. The reduced number of osteoclasts after ARQ-197 treatment may also have had a role to play in the reduced tumour volume as osteoclasts have been found to stimulate proliferation and survival of MM cells [52]. This supports previous findings on human bone biopsies from myeloma patients, which showed increased erosion depth with increased plasma cell infiltration [53]. Treatment with ARQ-197 reduced tumour volume and therefore reduced osteoclast numbers and increased osteoblast numbers. This effect was also reflected in the histological assessment of bone formation. Tumour-bearing mice had reduced bone formation compared to naïve mice and this reduction was inhibited after treatment with ARQ-197. We also tested the efficacy of ARQ-197 on naïve mice with no tumour load. Compared to naïve mice receiving no treatment, the ARQ-197 treated naïve mice showed no significant changes in osteoblast number, osteoclast number or bone formation indices. Taken together, these results imply that the inhibition of bone loss in tumour-bearing mice treated with ARQ-197 is a consequence of tumour load reduction, and the prevention of HGF-induced inhibition of osteoblasts, rather than a direct bone anabolic effect.

In summary, this study showed that ARQ-197 inhibited proliferation and induced death in the JJN3 human myeloma cell line, and reduced tumour volume and inhibited bone loss in a murine model of MM. Collectively, the findings generated in this study show ARQ-197 may be an effective anti-tumour agent in the treatment of MM as others have shown, but more importantly may also prevent myeloma-induced bone loss both as an indirect consequence of reduced tumour load and as a direct consequence of blockade of HGF induced inhibition of bone formation. Further studies are now required to evaluate its potential in combination therapy, possibly in tandem with zoledronic acid and standard chemotherapy regimens.

## Supporting information

S1 Fig ARQ-197 does not affect trabecular bone fraction in naïve mice. **(A)** Trabecular bone fraction (BV/TV %) of the tibiae or **(B)** vertebrae (L3) analysed by μCT from naïve mice (Naïve) and naïve mice treated with ARQ-197 (Naïve + ARQ-197). All data displayed as mean ± SD and analysed using an unpaired t-test.
(TIF)

S2 Fig ARQ-197 has no effect on osteoclastic bone resorption on cortico-endosteal surfaces of the tibiae from naïve mice. **(A)** Histomorphometric analysis of the number of TRAP positive osteoclasts per mm cortico-endosteal bone (N.Oc/BS,mm) from naive mice (Naïve) and naïve mice treated with ARQ-197 (Naïve + ARQ-197). **(B)** The percentage coverage of TRAP positive osteoclasts on the cortico-endosteal bone (Oc.S/BS (%) from naive mice (Naïve) and naive mice treated with ARQ-197 (Naive+ARQ-197). All data displayed as mean ± SD and analysed using an unpaired t-test.
(TIF)

S3 Fig ARQ-197 has no effect on osteoblasts on cortico-endosteal surfaces of the tibiae from naïve mice. **(A)** Histomorphometric analysis of the number of osteoblasts per mm cortico-endosteal bone (N.Ob/BS,mm) from naive mice (Naïve) and naïve mice treated with ARQ-197 (Naïve + ARQ-197). **(B)** The percentage coverage of osteoblasts on the cortico-endosteal bone (Ob.S/BS (%) from naive mice (Naïve) and naive mice treated with ARQ-197 (Naive+ARQ-197). All data displayed as mean ± SD and analysed using an unpaired t-test.
(TIF)

S4 Fig ARQ-197 has no effect on bone formation on the cortico-endosteal surface of the tibiae from naïve mice. **(A)** Histomorphometric analysis of the mineralising surface (MS, %) **(B)** the mineral apposition rate (MAR, μm/day) and **(C)** the bone formation rate (BFR/BS, mm^2^ X 10^−3^/mm/day) on the cortico-endosteal bone surface of tibiae from naive mice (Naïve) and naïve mice treated with ARQ-197 (Naïve + ARQ-197). All data displayed as mean ± SD and analysed using an unpaired t-test.
(TIF)

S5 Fig Full western blot from Fig 1. (TIF)

S6 Fig Full western blot of phospho c-Met from Fig 2. (TIF)

S7 Fig Full western blot of c-Met from Fig 2. (TIF)

S1 Table HGF expression data for myeloma cell lines in Fig 1. (XLSX)

S2 Table Relative density values from western blot in Fig 1. (XLSX)

S3 Table Cell death and cell proliferation data from Fig 2. (XLSX)

S4 Table Tumour, Ki-67 and Annexin V counts from Fig 3. (XLSX)

S5 Table uCT values from Fig 4. (XLSX)

S6 Table Histomorphometry data from Fig 5. (XLSX)

S7 Table Histomorphometry data from Fig 6. (XLSX)

S8 Table Histomorphometry data from Fig 7. (XLSX)

S9 Table uCT values from S1 Fig. (XLSX)

S10 Table Histomorphometry data from S2, S3 and S4 Figs. (XLSX)
