# Supplementary figures and images for: ARQ-197, a small-molecule inhibitor of c-Met, reduces tumour burden and prevents myeloma-induced bone disease in vivo
Source: PLoS One. 2018 Jun 20;13(6):e0199517. doi: 10.1371/journal.pone.0199517 (PMC6010293; doi:10.1371/journal.pone.0199517)

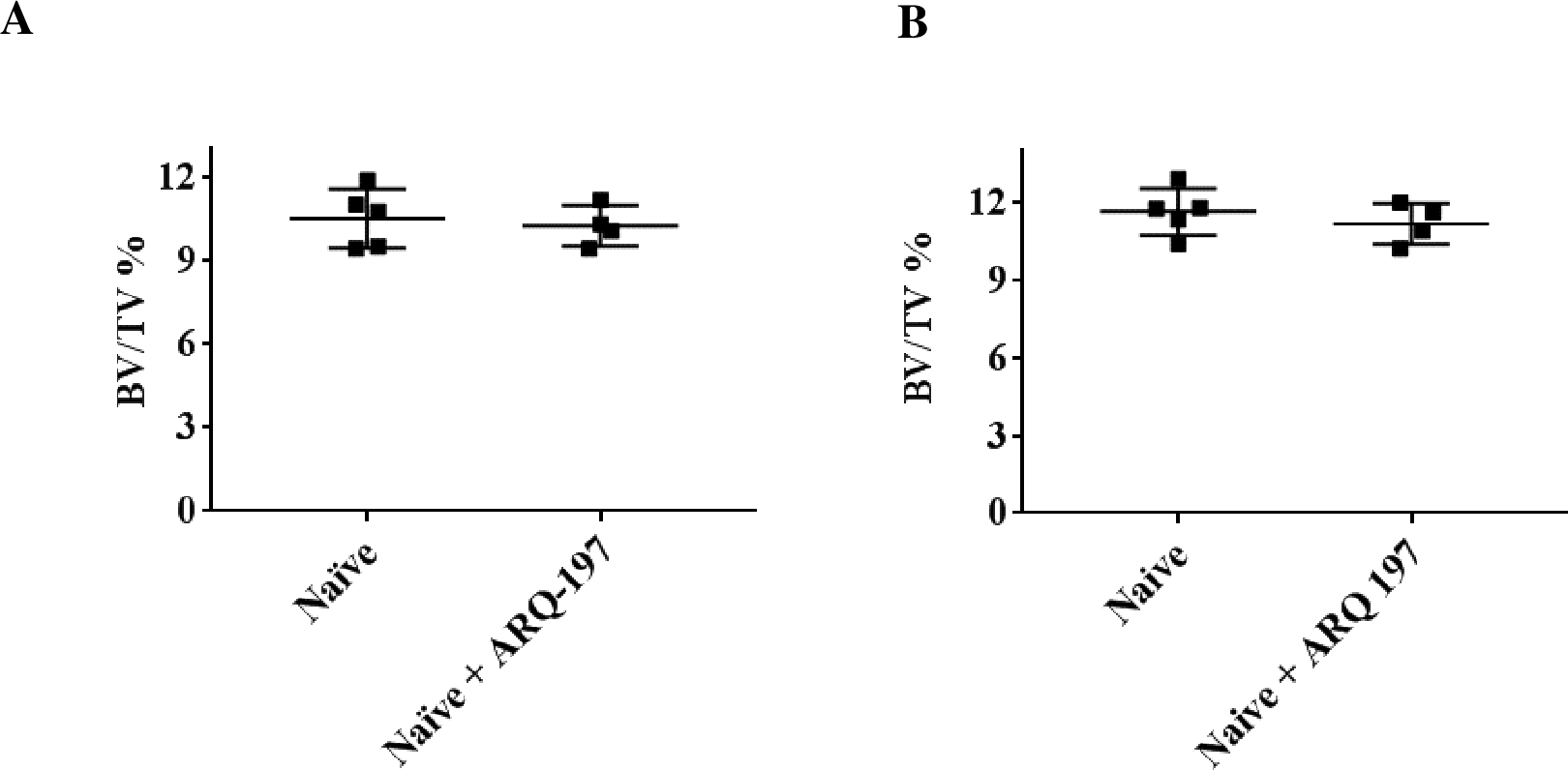

Supplement: S1 Fig — (A) Trabecular bone fraction (BV/TV %) of the tibiae or (B) vertebrae (L3) analysed by μCT from naïve mice (Naïve) and naïve mice treated with ARQ-197 (Naïve + ARQ-197). All data displayed as mean ± SD and analysed using an unpaired t-test. (TIF) [file pone.0199517.s001.tif]

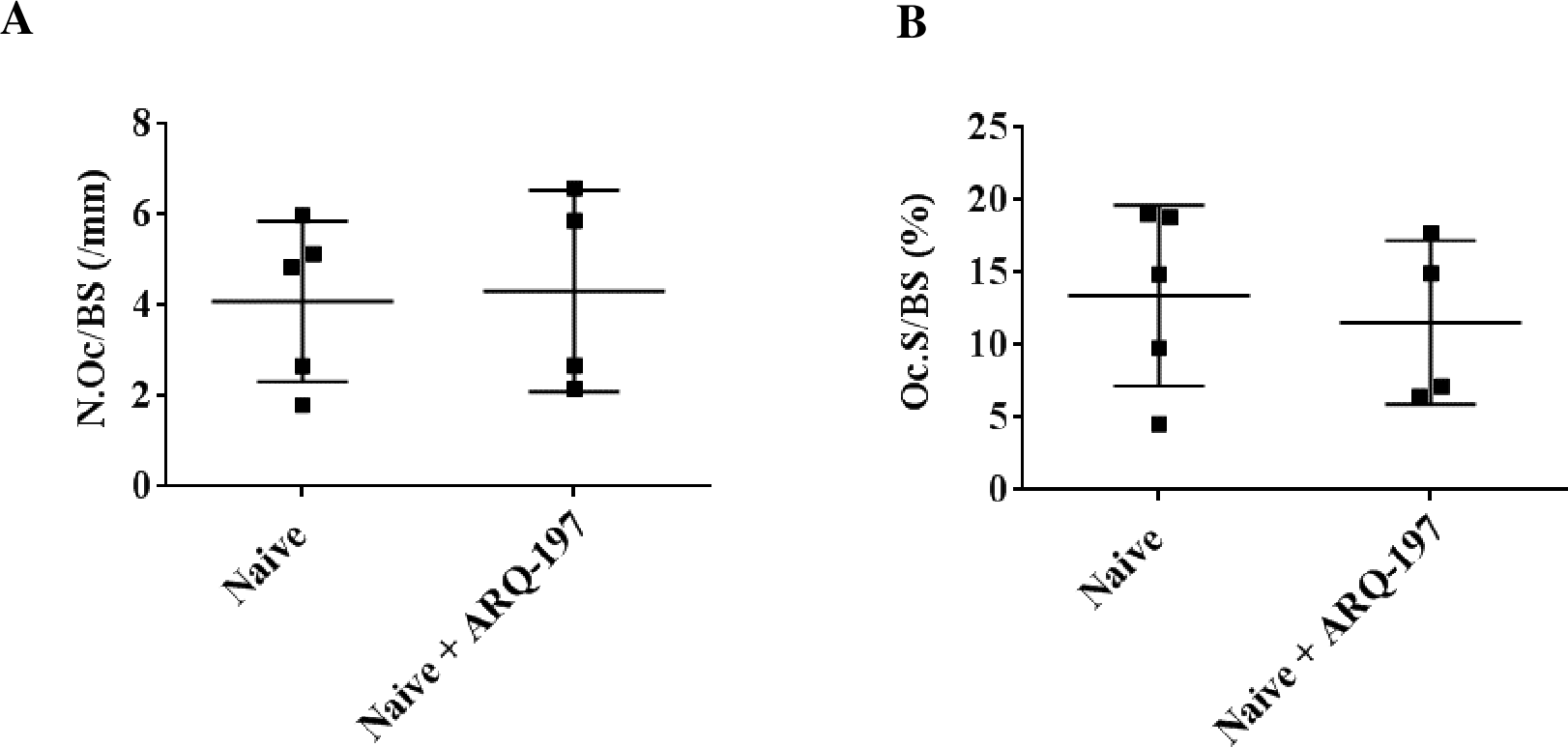

Supplement: S2 Fig — (A) Histomorphometric analysis of the number of TRAP positive osteoclasts per mm cortico-endosteal bone (N.Oc/BS,mm) from naive mice (Naïve) and naïve mice treated with ARQ-197 (Naïve + ARQ-197). (B) The percentage coverage of TRAP positive osteoclasts on the cortico-endosteal bone (Oc.S/BS (%) from naive mice (Naïve) and naive mice treated with ARQ-197 (Naive+ARQ-197). All data displayed as mean ± SD and analysed using an unpaired t-test. (TIF) [file pone.0199517.s002.tif]

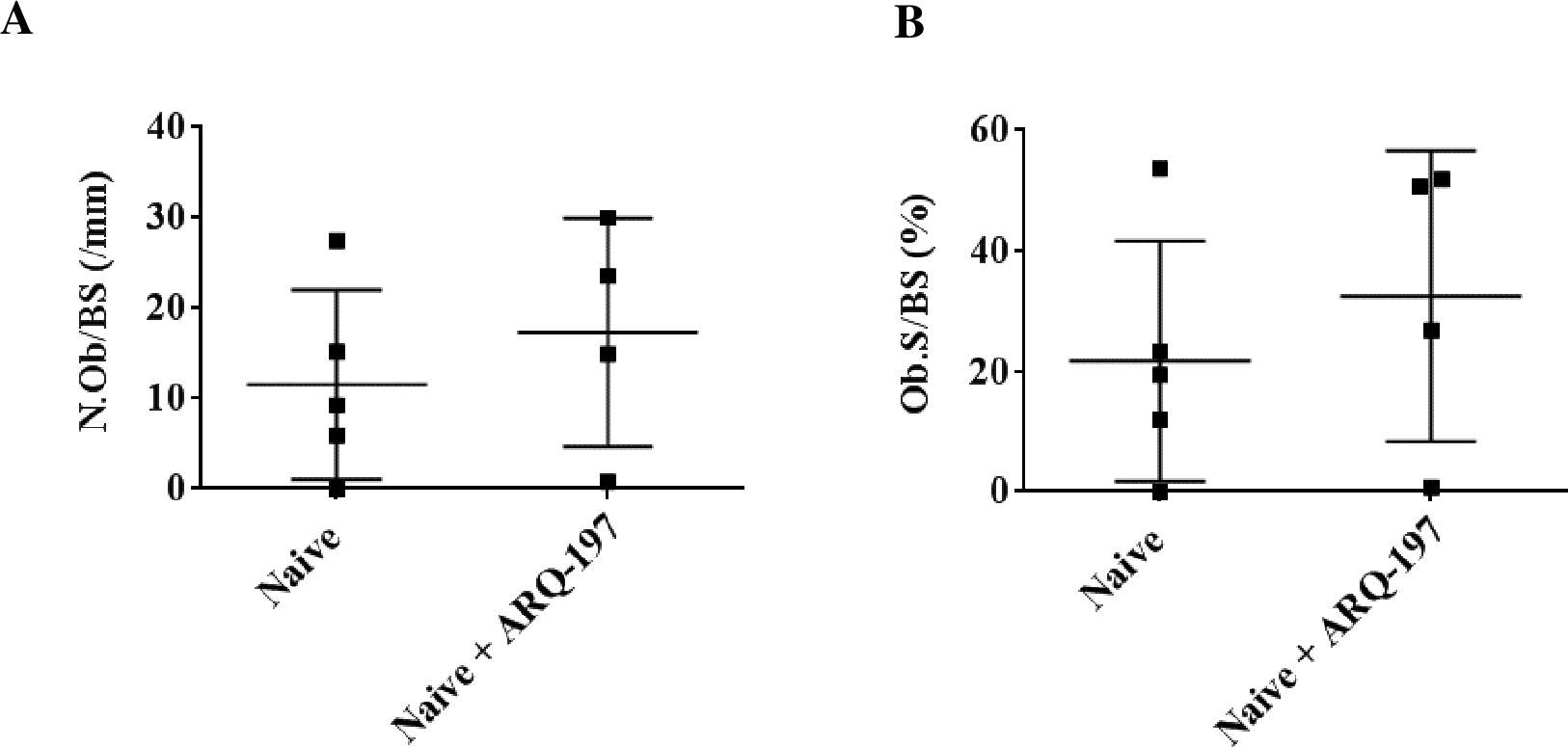

Supplement: S3 Fig — (A) Histomorphometric analysis of the number of osteoblasts per mm cortico-endosteal bone (N.Ob/BS,mm) from naive mice (Naïve) and naïve mice treated with ARQ-197 (Naïve + ARQ-197). (B) The percentage coverage of osteoblasts on the cortico-endosteal bone (Ob.S/BS (%) from naive mice (Naïve) and naive mice treated with ARQ-197 (Naive+ARQ-197). All data displayed as mean ± SD and analysed using an unpaired t-test. (TIF) [file pone.0199517.s003.tif]

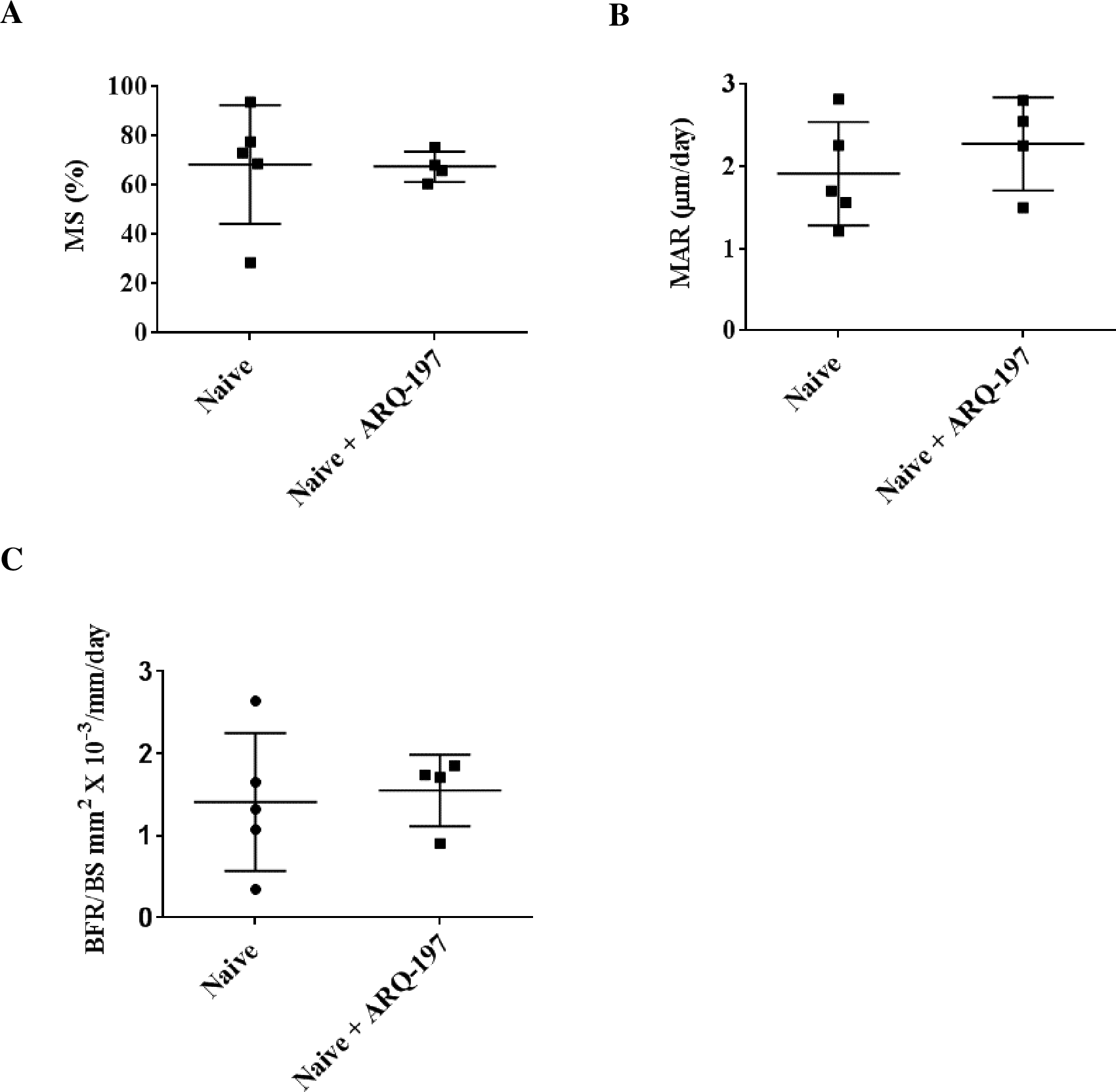

Supplement: S4 Fig — (A) Histomorphometric analysis of the mineralising surface (MS, %) (B) the mineral apposition rate (MAR, μm/day) and (C) the bone formation rate (BFR/BS, mm2 X 10−3/mm/day) on the cortico-endosteal bone surface of tibiae from naive mice (Naïve) and naïve mice treated with ARQ-197 (Naïve + ARQ-197). All data displayed as mean ± SD and analysed using an unpaired t-test. (TIF) [file pone.0199517.s004.tif]

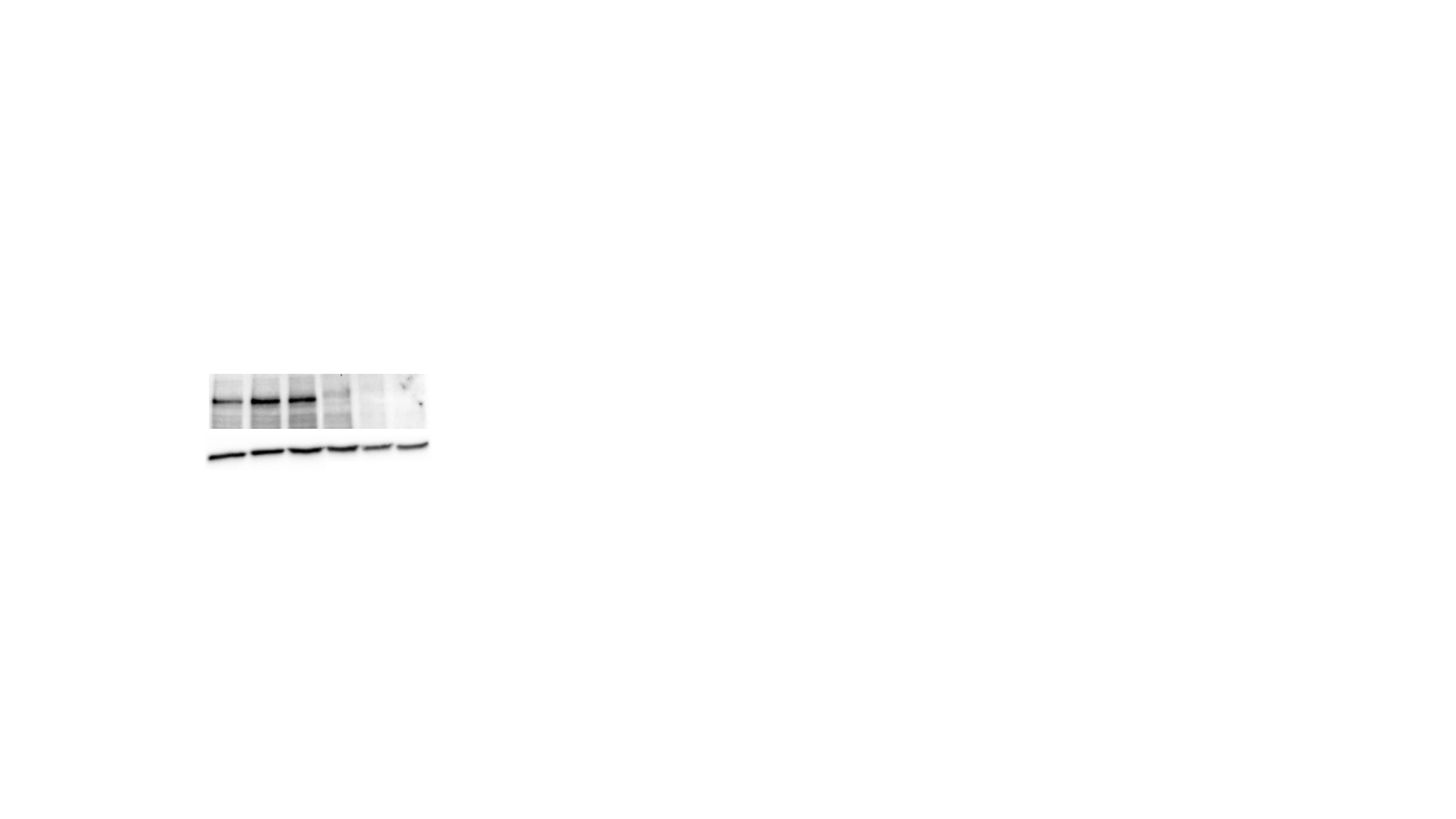

Supplement: S5 Fig — (TIF) [file pone.0199517.s005.tif]

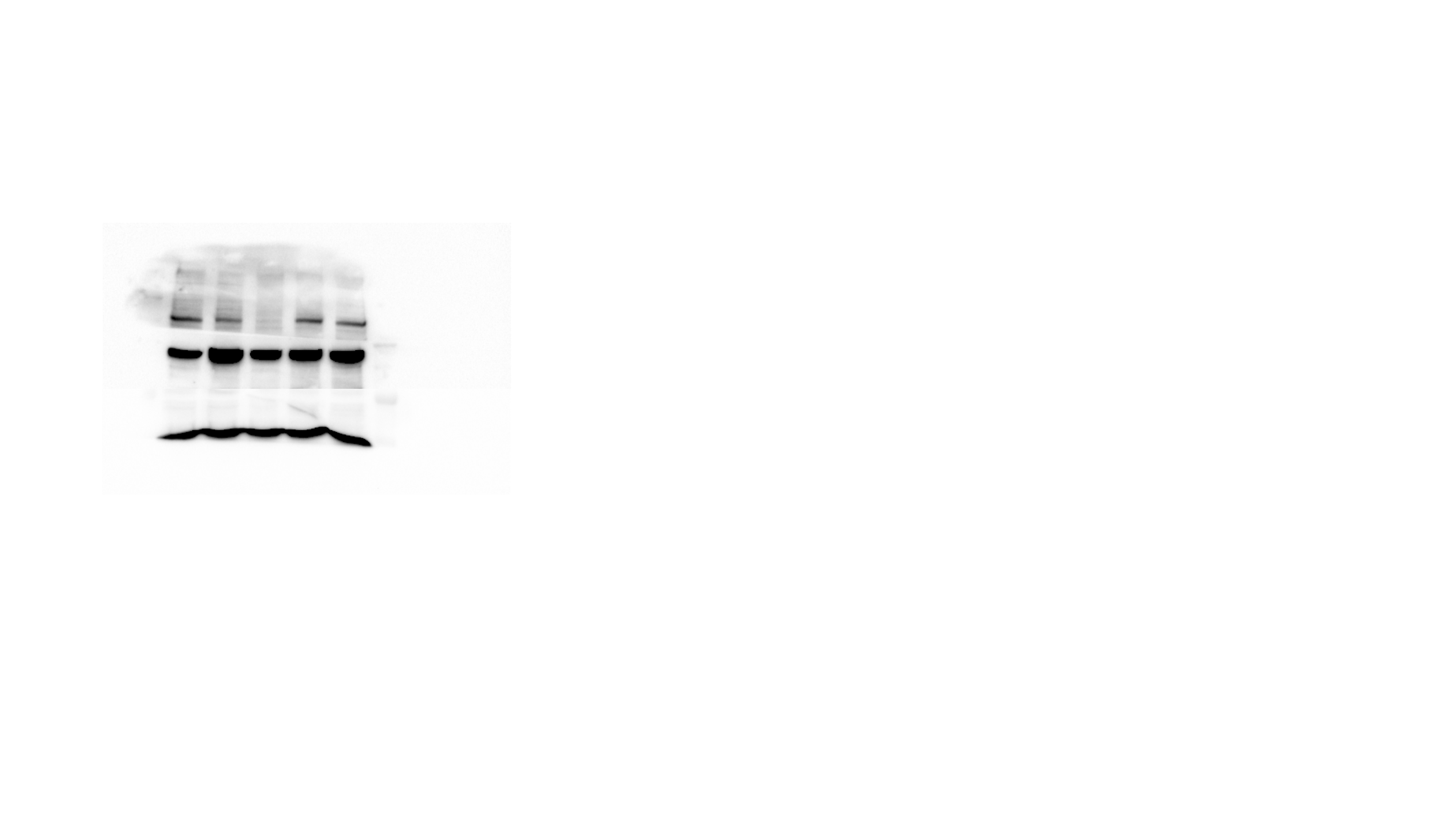

Supplement: S6 Fig — (TIF) [file pone.0199517.s006.tif]

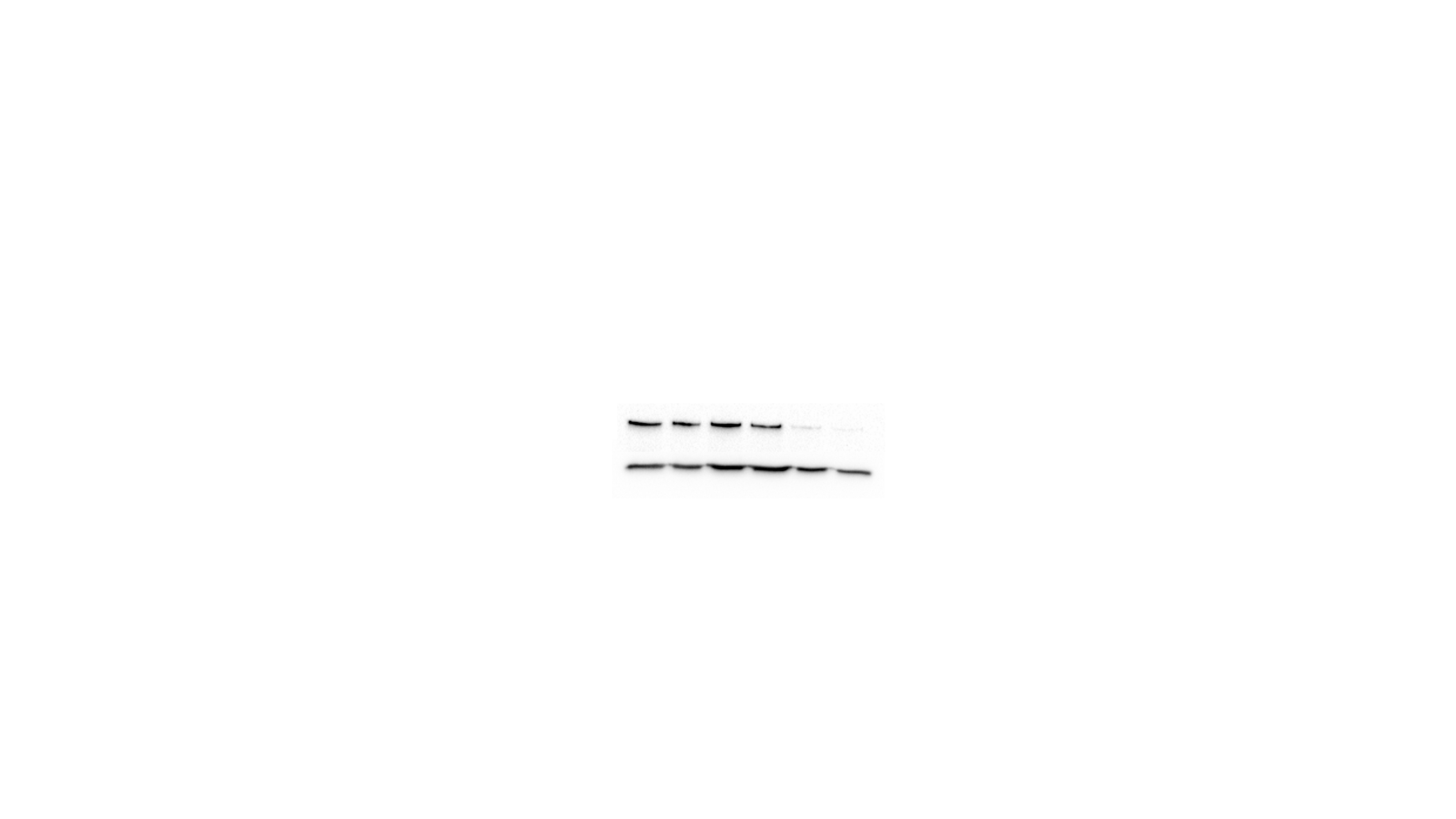

Supplement: S7 Fig — (TIF) [file pone.0199517.s007.tif]
